# Evaluation of *In Vitro* Uterotonic Activities of Fruit Extracts of *Ficus asperifolia* in Rats

**DOI:** 10.1093/ecam/nep221

**Published:** 2011-03-13

**Authors:** Pierre Watcho, Esther Ngadjui, Pepin Alango Nkeng-Efouet, Telesphore Benoît Nguelefack, Albert Kamanyi

**Affiliations:** ^1^Animal Physiology and Phytopharmacology Laboratory, Department of Animal Biology, Faculty of Science, University of Dschang, Po Box 377 Dschang, Cameroon; ^2^Department of Chemistry, University of Dschang, Box 67 Dschang, Cameroon

## Abstract

The aim of the present study was to determine the uterotonic activities of *Ficus asperifolia* and investigate its mechanism. The effects of aqueous and methanol extracts of the dried fruits of *F. asperifolia* (0.05–1.60 mg mL^−1^) were evaluated on estrogenized isolated rat uterus in the presence and absence of atropine (1.73–55.27 nM), pyrilamine maleate (1.25 × 10^−3^ to 40 × 10^−3^ M), indomethacin (0.06 × 10^−5^ to 2.00 × 10^−5^ M) or hexamethonium (0.66 × 10^−4^ to 21.43 × 10^−4^ M). Aqueous (EC_50_, 0.36 mg mL^−1^) and methanol (EC_50_, 0.22 mg mL^−1^) extracts as well as oxytocin (EC_50_, 0.02 nM), acetylcholine (EC_50_, 7.87 nM) and histamine (EC_50_, 0.76 nM) evoked concentration-dependent contractions of the uterus. Atropine, pyrilamine maleate and indomethacin concentration dependently blocked the response of the uterus to acetylcholine (IC_50_, 4.82 nM), histamine (IC_50_, 2.49 nM) and oxytocin (IC_50_, 0.07 nM), respectively, and to aqueous extract. Hexamethonium produced graded decreases in oxytocin-induced uterine contractions (IC_50_, 0.37 **μ**M), but did not prevent the contractile effects of the aqueous extract (IC_50_, 9.88 **μ**M). These results suggest that *F. asperifolia*-induced uterotonic effect is related to the release of prostaglandins and contraction of the myometrial cells through muscarinic, oxytocic and H_1_ histamine receptors. These data further give added value to the ethnic use of *F. asperifolia* for its abortificient and contraceptive properties.

## 1. Introduction

The world population is estimated to reach 8.9 billion by the year 2050, far higher than the 2004 estimate of 6.4 billion [1, 2]. Population control is therefore needed for individual and national welfare. Several effective contraceptive methods exist and are used for fertility control among countries and regions of the world. In the western societies, the pattern use of female contraceptives varies from hormonal (steroidal and non-steroidal molecules), surgical (sterilization) to mechanical techniques (intrauterine devices, condom) [3]. In the developing countries where modern contraceptive options are limited, >80% of the population continues to use medicinal plants as extract, powder or decoction to prevent unintended pregnancy or in family planning. Plants like *Hibiscus rosa-sinensis*, *Calotropis gigantean*, *Cissampelos pareira* and many others have been reported to exhibit some contraceptive potentials [4–7]. *Ficus asperifolia* belonging to the *Moraceae* family is also one of such plants. It is a small or average size tree, terrestrial or epiphyte which can reach 20 m in height. It is found in Senegal, Uganda, Tanzania, Natal (South Africa), Madagascar and Cameroon. *F. asperifolia* is abundant in the savannah regions, especially along river banks and marshy areas at an altitude of up to 1100 m. The leaves are enormous and displayed spirally, the limb is largely oval or has a form of ellipse and the roots are most often fibrous [8]. In many traditional medicines of Africa, the leaf extract of *F. asperifolia* is used as an anthelmintic and a purgative while the decoction of the dried fruits and the bark extract are reputed for its fertility regulation, abortificient and contraceptive properties [9]. However, these reports are still not scientifically investigated. The aim of the present study was therefore to assess the effects of *F. asperifolia* on the isolated uterine tissue and to propose its possible mechanism of action relevant to its popular use as an abortificient/contraceptive. We used the estrogenized isolated uterine model since it had been demonstrated that pre-treatment of females with estrogens increases the responsiveness of the isolated uterus to drugs exhibiting uterotonic activities [10–13].

## 2. Methods

### 2.1. Collection of Plant Material and Preparation of Extracts

Fresh fruits of *F. asperifolia* (L) Hook. ex Miq. (Moraceae) were collected in February 2006 from trees in Dschang, Cameroon. Botanical identification was performed in the Cameroon National Herbarium (HNC) where a voucher No. 338/15240/HNC has been deposited. The fruits were shade-dried for 5 days and ground into powder. Two types of extracts were used in the study. In order to obtain an aqueous extract similar to the traditional recommendation, 1 kg of *F. asperifolia* was soaked in distilled water (5 L) and the mixture boiled for 15 min. The heated decoction was taken and allowed to cool at room temperature, filtered using Whatman paper No. 3 and oven-dried to give 46.67 g of dried aqueous extract (yield of extraction, 4.66%; w/w based on the dried starting weight). To obtain the methanol extract, 1 kg of *F. asperifolia* powder was soaked in 5 L of methanol (95%) for 24 h. The extract was filtered using Whatman paper No. 3 and the filtrate was evaporated (78°C) to dryness using a rotary evaporator; 50 g of dried methanol extract was obtained giving an extraction yield of 5% (w/w based on the dried starting weight). For bioactivity investigations, the aqueous and methanol extracts were dissolved in distilled water and 5% Tween 80, respectively.

### 2.2. Preliminary Phytochemical Screening

The freshly prepared aqueous and methanol extracts were tested for the presence of chemical constituents employing standard procedures including the Libermann Buchard's test for the existence of sterols and triterpens, the test of Shinoda for the presence of flavonoids and the test for saponins as described by Hosttetmann et al. [14–17].

### 2.3. Animals

Healthy non pregnant adult female Wistar rats between 10 and 12 weeks of age and weighing 150–170 g were used in this study. They were housed in groups (five per group in polypropylene cages) and maintained under uniform husbandry conditions having natural photoperiod, humidity, temperature (26  ±  2°C) and free access to food and water. The Local Committee of Ethics on Animal Experimentation approved all experimental procedures, which followed the regulations established by the European Union on Animal Care and Experimentation (CEE Council 86/609).

### 2.4. Experimental Design

#### 2.4.1. Isolated Organ Preparation

To obtain the estrogenized uterus, virgin female rats were subcutaneously injected with 17-*β*-estradiol benzoate (13.28 nM per animal) and killed 24 h later by decapitation. The uteri were promptly removed, cleaned of the connective tissue and cut into strips of about 1 cm of length. Each uterine strip was mounted in an organ bath of 20 mL capacity containing fresh De Jalon solution of the following composition (mM): NaCl 153.85, KCl 5.64, CaCl_2_ 0.55, MgSO_4_ 0.08, NaOH 12.5 and glucose 2.78. The physiological salt solution (PSS) was maintained at 37  ±  0.50°C and continuously bubbled with air. The preparation was allowed to equilibrate for 30 min during which the bathing solution was changed every 10 min.

#### 2.4.2. Drug Challenges

After 30 min of equilibration period, uterine contractile responses were elicited by adding non-cumulatively acetylcholine (0.50–17.60 *μ*M), oxytocin (0.05–1.50 *μ*M), histamine (1.09–34.77 *μ*M), aqueous or methanol extracts of *F. asperifolia* (0.05–1.60 mg mL^−1^) to the De Jalon solution. Each dose of the drugs was allowed to act for 10 min and the amplitude of contraction recorded. The contractions were recorded by means of an isotonic transducer (Ugo Basile, Italy) connected to a single channel recorder (Ugo Basile, Italy) which was calibrated to record change in the tension generated on g versus cm displacement basis. The tension applied to the preparation was 0.71 g. Atropine (1.73–55.27 nM), indomethacin (0.06 × 10^−5^ to 2.00 × 10^−5^ M), pyrilamine maleate (1.25 × 10^−3^ to 40 × 10^−3^ M) and hexamethonium (0.66 × 10^−4^ to 21.43 × 10^−4^ M) were used to antagonize in a concentration-dependent manner the maximal response of the isolated uterus to acetylcholine (0.50–17.60 *μ*M), oxytocin (0.05–1.50 *μ*M) or histamine (1.09–34.77 *μ*M) and to the aqueous extract of *F. asperifolia* (1.60 mg mL^−1^), the most efficient extract.

#### 2.4.3. Drugs

The following drugs of analytical grade quality were purchased from Sigma Chemical (St Louis, MO, USA): estradiol benzoate (17-*β*-diol 3-benzoate), acetylcholine hydrochloride [ethanaminium, 2-(acetyloxy)-*N,N,N*-trimethyl-, chloride], oxytocin (*α*-eypophamine), histamine [2-(4-imidazolyl)ethylamine dihydrochloride], indomethacin [1-(4-chlorobenzyl)-5-methoxy-2-methyl-3-indoleacetic acid], pyrilamine maleate [*N*-(4-methoxyphenyl) methyl-*N*′] and hexamethonium chloride (*N,N,N,N*′,*N*′,*N*′-hexamethyl-1,6-hexanediaminium dichloride). Atropine sulfate [*α*-(hydromethyl) benzeneacetic 8-methyl-8-azabicyclo (3.2.1) oct-3-yl-ester] was purchased from a local pharmacy.

#### 2.4.4. Statistical Analysis

Data are expressed in mean  ±  SEM. One-way analysis of variance (ANOVA) followed by *post hoc* Newman–Keuls were used to analyze statistical difference among groups. In all experiments, the contractile responses were expressed as a percentage of the maximal contractile response to a reference drug. EC_50_ or IC_50_ values were calculated using GraphPad Prism version 3.00 for Windows, GraphPad Software, San Diego, CA, USA, http://www.graphpad.com/. A probability of *P* < .05 was accepted as significant.

## 3. Results

### 3.1. Preliminary Phytochemical Analysis

The fresh aqueous and methanol extracts of *F. asperifolia* gave a positive reaction to alkaloids, saponins, sterols and triterpens.

### 3.2. F. asperifolia and Agonist-Induced Uterine Contraction

**Figure 1:**
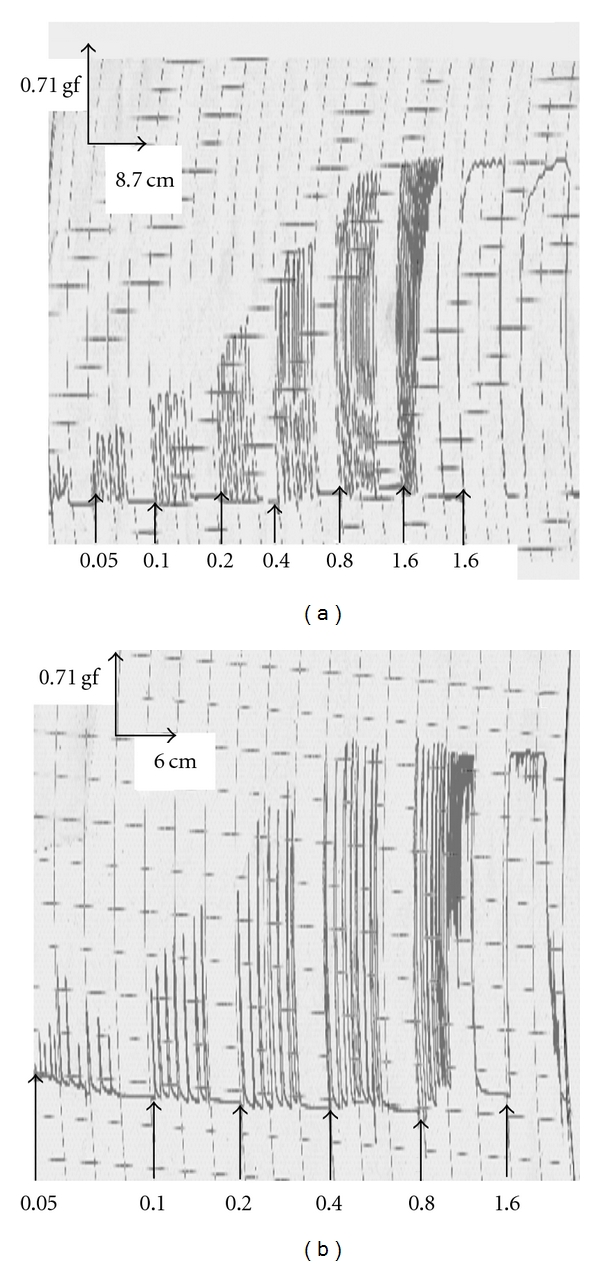
Representative traces showing the contractile activity of the isolated estrogenized uterus by the aqueous (b) and methanol (b) extracts of *F. asperifolia* (0.05–1.6 mg/ml). Between two consecutive concentrations (indicated by the arrows), the tissue was allowed to equilibrate for 10–15 min during which the uterus was washed at least two times.

**Figure 2:**
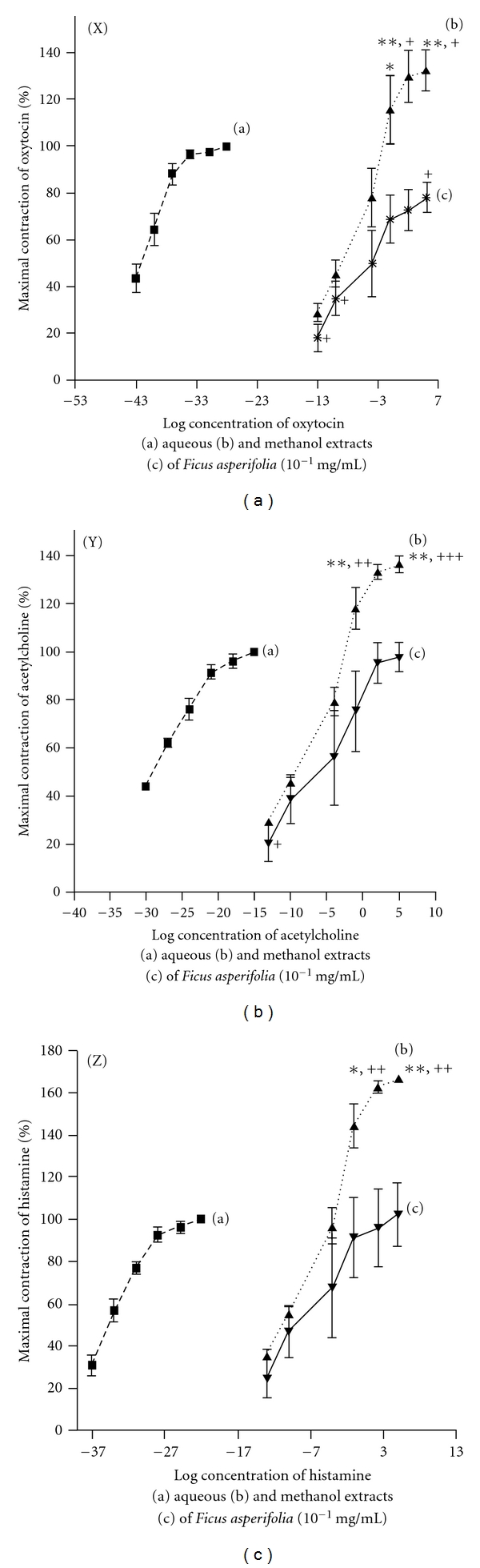
Comparative mean concentration-response curves for aqueous and methanol extracts of *F. asperifolia* with oxytocin (X), acteylcholine (Y) and histamine (Z). Each point represents mean of four experiments.  *P < .05;  **P < .01 significant when compared to methanol extract.  ^+^ *P* < .05;  ^++^ *P* < .01;  ^+++^ *P* < .001 significant when compared to standard agonist.

Aqueous and methanol extracts of the dried fruits of *F. asperifolia* evoked concentration-dependent contractions of the rat uterine smooth muscle. In most preparations, phasic contractions were developed at lower concentrations (0.05–0.40 mg mL^−1^) while a strong tonic contraction was usually obtained with the concentration 1.60 mg mL^−1^ (Figure 1). Acetylcholine (0.50–17.60 *μ*M), oxytocin (0.05–1.50 *μ*M) and histamine (1.09–34.77 *μ*M) also provoked contractions of the uterine tissue with oxytocin being the most potent (EC_50_, 0.02 nM) compared to acetylcholine (EC_50_, 7.87 nM) and histamine (EC_50_, 0.76 nM). With reference to the percentage of maximal amplitude of contraction induced by the standard agonists used in this study, the aqueous extract (EC_50_, 0.36 mg mL^−1^) of *F. asperifolia* always increased (*P* < .05 − .001) the amplitude of contraction of the uterine smooth muscle at high concentrations (0.80–1.60 mg mL^−1^) whilst a tendency to low effects was observed in the case of methanol extract (EC_50_, 0.22 mg mL^−1^) (Figure 2).

### 3.3. Antagonist Studies

**Figure 3:**
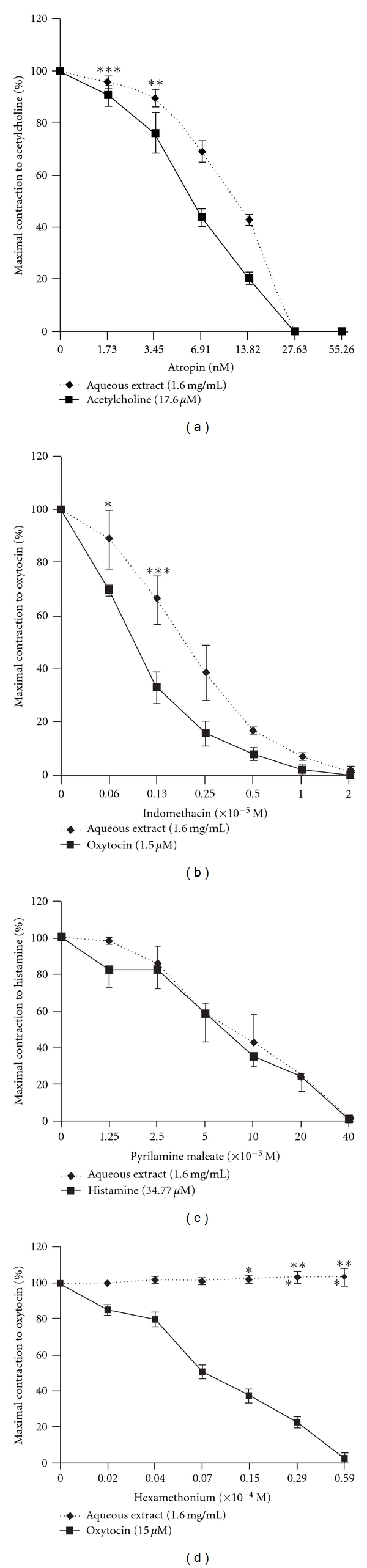
Concentration-dependent effects of atropine (a), indomethacin (b), pyrilamine maleate (c) and hexamethonium (d) on the maximum contractile response of the isolated estrogenized uterus to the aqueous extract of *F. asperifolia* (1.6 mg/mL). Acetylcholine, oxytocin and histamine were used as standard agonists. Atropin, pyrilamine maleate and hexamethonium were administered 10 min before addition of plant extract or standard agonists. Indomethacin was introduced 20 min before addition of oxytocin or aqueous extract. Each bar represents mean of four experiments.  *P < .05;  **P < .01;  ***P < .001 significant when compared to standard agonist.

The evidence that aqueous extract of *F. asperifolia* (EC_50_, 0.36 mg kg^−1^), oxytocin (EC_50_, 0.02 nM), acetylcholine (EC_50_, 7.87 nM) and histamine (EC_50_, 0.76 nM) concentration dependently induced contractions of the estrogenized rat uterus was clearly shown in the present study (Figures 1 and 2). Atropine (1.73–55.27 nM) which is a non-specific muscarinic competitive antagonist reduced in a concentration-dependent way the maximal contractile responses to acetylcholine (IC_50_, 4.82 nM) and aqueous extract of *F. asperifolia* (IC_50_, 12.58 nM) (Figure 3(a)). Pre-treatment of uterine horns with pyrilamine maleate (1.25 × 10^−3^ to 40 × 10^−3^ M), a H_1_ histamine receptor antagonist, progressively and almost equally abolished the contractile effects of both histamine (IC_50_, 2.49 nM) and aqueous extract of *F. asperifolia* (IC_50_, 2.49 nM) (Figure 3(c)). Indomethacin (0.06 × 10^−5^ to 2.00 × 10^−5^ M), an inhibitor of prostaglandin synthase, suppressed both oxytocin (IC_50_, 0.07 nM) and aqueous extract (IC_50_, 1.17 nM)-induced uterine contractions (Figure 3(b)). Hexamethonium (0.66 × 10^−4^ to 21.43 × 10^−4^ M), a ganglionic blocking agent, did not affect the contractile responses induced by the aqueous extract (IC_50_, 9.88 *μ*M) but produced graded decreases in oxytocin-induced uterine contractions (IC_50_, 0.37 *μ*M) (Figure 3(d)). It is noteworthy mentioning that pyrilamine maleate similarly abolished the contractile effects of the aqueous extract of *F. asperifolia* (1.60 mg mL^−1^) and histamine (34.77 *μ*M), whereas rightward shift of the concentration-response curves to atropine and indomethacin was observed.

## 4. Discussion and Conclusions

**Figure 4:**
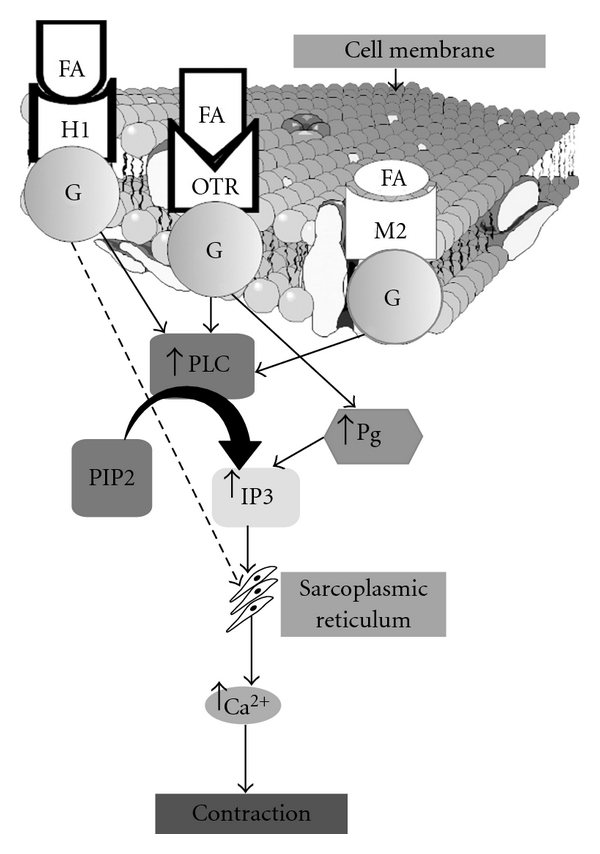
Schematic diagram of proposed mechanism of *F. asperifolia*-induced contraction in estrogen-treated uterine muscle. For detailed description, see Discussion section. FA: *Ficus asperifolia*; H1: type 1 histamine receptor; OTR: oxytocin receptor; M2: type 2 muscarinic receptor; G: protein G; PLC: phospholipase C; Pg: prostaglandin; PIP2: phosphatidylinositol 4,5 biphosphate; IP3: inositol 1,4,5 triphosphate.

Results of the present study demonstrated the oxytocic-like activities of *F. asperifolia* in the estrogenized isolated rat uterus. The aqueous and methanol extracts produced a concentration-dependent increase in contraction of the estrogenized isolated rat uterus with the aqueous extract (EC_50_, 0.36 mg mL^−1^) exhibiting the high efficacy although the methanol extract likely showed greater potency (EC_50_, 0.22 mg mL^−1^). A great number of medicinal plants are also known for their oxytocic potentials [10–13]. With regard to the major concern of the study, data of this work further highlight the uterotonic claimed activity of this medicinal plant and which could mimic that of some uterine contractile agents. With respect to the concentration-dependent contractile responses evoked by acetylcholine, histamine and oxytocin, many studies have indicated the existence of abundant cholinergic receptors in the uterine smooth muscle and that stimulation of myometrial muscarinic receptors by agonists such as acetylcholine causes contraction of the uterus [18, 19]. It is also well established that histamine receptors (H_1_ and H_2_) are present in the uterus and the predominant response of histamine in this tissue is contraction (H_1_ activity) [20]. Oxytocin, the most potent of the endogenous oxytocics, acts on myometrial oxytocin receptors (OT_1a_) to directly cause uterine contraction and on endometrial oxytocin receptors (OT_1b_) to stimulate prostaglandins and cholinergic releases leading to uterine contraction [21–25]. Phospholipase C-mediated mobilization of mainly sarcoplasmic intracellular calcium via inositol triphosphate is the major intracellular mechanism after oxytocin, histamine and acetylcholine initiate signal transduction by binding to G protein-coupled receptor in the cell membrane (Figure 4) [26–28].

In order to ascertain the involvement of cholinergic, histaminergic and oxytocinergic pathways in the mechanism of *F. asperifolia*-induced uterine contractions, the effect of the most active plant extract (aqueous extract) was evaluated in the presence of adequate antagonists of these substances. Thus, pre-treatment of uterine strips with atropine (1.73–55.27 nM), indomethacin (0.06 × 10^−5^ to 2.00 × 10^−5^ M) or pyrilamine maleate (1.25 × 10^−3^ to 40 × 10^−3^ M) antagonised concentration dependently the maximal response to the plant extract (1.60 mg mL^−1^) and to the respective agonists namely acetylcholine, oxytocin and histamine in this order. The rightward shift curves observed in the presence of atropine and indomethacin probably suggest partial agonistic effects of the aqueous extract of *F. asperifolia* to acetylcholine and oxytocin, respectively. The fact that pyrilamine maleate equally inhibited the contractile effects of *F. asperifolia* and histamine indicates that *F. asperifolia* especially it aqueous extract could be considered as a full agonist to histamine (H_1_). These results imply that the bioactive compounds found in the aqueous extract of *F. asperifolia* appear to activate the endometrial and myometrial cell membrane receptors resulting in an uterotonic effect by a mode of action possibly via the prostaglandin synthesis and the activation of cholinergic, oxytocic and/or histamine receptors. Phytochemical screenings of the aqueous and methanol extract of *F. asperifolia* have revealed the presence of alkaloids and saponins. Data from the literature indicate that these two well-known compounds possess uterine stimulating effects [29–31]. It could therefore be understood that the presence of these biological principles in the extracts of *F. asperifolia,* especially its aqueous extract, may account for the observed uterine contractile activity. The involvement of muscarinic, oxytocic and H_1_ receptors was also found to mediate uterine muscle contractility in response to some plant extracts [11, 12, 23, 24]. Of great interest in this study is the lack of effect of the ganglionic blocking agent, hexamethonium, on the contractile action of the aqueous extract of *F. asperifolia*; this finding seems to indicate that the crude aqueous extract-induced increase in myometrial contractility is due to actions on post-ganglionic autonomic nerve endings with involvement of many receptors and further supports the hypothesis of an oxytocic-like potential of the plant extract [19].

In conclusion, our data provide evidence to suggest that *F. asperifolia*-induced uterotonic effect in the estrogenized isolated rat uterus is owing to a mechanism related to the release of prostaglandins and contraction of the myometrial cells through more than one mechanism including the muscarinic, oxytocic and histamine receptors. These findings justify the traditional use of the plant for its abortificient and contraceptive properties. However, in the absence of a specific antagonist to oxytocin, it was not possible to characterize the direct implication of oxytocic receptors in the effects of *F. asperifolia*. Further studies are therefore needed to clearly elucidate the mode of contraceptive action of this medicinal plant.
